# Factors associated with failure of bony union after conservative treatment of acute cases of unilateral lumbar spondylolysis

**DOI:** 10.1186/s12891-020-03940-9

**Published:** 2021-01-13

**Authors:** Masaki Tatsumura, Hisanori Gamada, Shun Okuwaki, Fumihiko Eto, Katsuya Nagashima, Takeshi Ogawa, Takeo Mammoto, Atsushi Hirano, Masao Koda, Masashi Yamazaki

**Affiliations:** 1 Department of Orthopaedic Surgery and Sports Medicine, Tsukuba University Hospital Mito Clinical Education and Training Center, Mito Kyodo General Hospital, 3-2-7 Miyamachi, 310-0015 Mito, Ibaraki Japan; 2 Department of Orthopaedic Surgery, Faculty of Medicine, University of Tsukuba, Tsukuba, Japan

**Keywords:** Lumbar spondylolysis, Union inhibitor, Contralateral pseudarthrosis lesion, Treatment failure and risk factor

## Abstract

**Background:**

If bone union is expected, conservative treatment is generally selected for lumbar spondylolysis. However, sometimes conservative treatments are unsuccessful. We sought to determine the factors associated with failure of bony union in acute unilateral lumbar spondylolysis with bone marrow edema including contralateral pseudarthrosis.

**Methods:**

This study targeted unilateral lumbar spondylolysis treated conservatively in high school or younger students. Conservative therapy was continued until the bone marrow edema disappeared on MRI and bone union was investigated by CT. We conducted a univariate analysis of sex, age, pathological stage, lesion level complicating the contralateral bone defect, lesion level, and intercurrent spina bifida occulta, and variables with *p* < 0.1 were considered in a logistic regression analysis. An item with *p* < 0.05 was defined as a factor associated with failure of bony union.

**Results:**

We found 92 cases of unilateral spondylolysis with bone marrow edema and 66 cases were successfully treated conservatively. Failure of bony union in unilateral lumbar spondylolysis with bone marrow edema was associated with progressive pathological stage (*p* = 0.004), contralateral pseudarthrosis (*p* < 0.001), and L5 lesion level (*p* = 0.002). The odds ratio was 20.0 (95% CI 3.0–193.9) for progressive pathological stage, 78.8 (95% CI 13–846) for contralateral pseudarthrosis, and 175 (95% CI 8.5–8192) for L5 lesion level.

**Conclusions:**

Conservative therapy aiming at bony union is contraindicated in cases of acute unilateral spondylolysis when the pathological stage is progressive, the lesion level is L5, or there is contralateral pseudarthrotic spondylolysis.

## Background

Lumbar spondylolysis is a fatigue fracture of pars interarticularis, most of which occur in the 5th lumbar spinal vertebra (L5) [1]. It often occurs in athletes and adolescents and can be fused by conservative treatment [2]. The union rate of lumbar spondylolysis is lower than that of general fatigue fractures. A wide variation in union rate has been reported: 87% in early stage spondylolysis [2], and < 56% despite treatment for up to 6 months or more [3]. A possible reason for the substantial variation in union rate between the reports is that there are multiple confounding factors that have a significant impact on bone union. Previous reports revealed several possible factors affecting bone union after conservative treatments for lumbar spondylolysis, including vertebral level [4], stage [4], contralateral condition [4], bilateral spondylolysis [5], and latent spina bifida [6, 7]. Contralateral pseudarthrosis might be an inhibitory factor for bony union because new spondylolysis tends to occur when a bone defect occurs on the opposite side of the vertebral arch [8]. However, the precise influence of contralateral pseudarthrosis on bony union rate remains to be elucidated.

Treatment strategies for lumbar spondylolisthesis are controversial. Especially for unilateral surgical treatment, it has been reported that surgery is desirable when symptoms persist for 6 months or longer [9]. In addition, even when surgical treatment is selected, if unilateral pars cleft is present, the surgical results are better than bilateral [10]. On the other hand, if bone fusion can be expected, conservative treatment is also an option. Where bony union cannot be expected by conservative therapy, symptomatic therapy or surgical therapy should be considered rather than continuing exercise or depriving patients of exercise opportunities. Therefore, it is necessary to establish a prognosis of bony union at the time of initial diagnosis of patients with lumbar spondylolysis to determine the treatment strategy. We sought to elucidate the major factors associated with failure of bony union in acute unilateral lumbar spondylolysis with bone marrow edema including contralateral pseudarthrosis.

## Patients and methods

### Patients

This study targeted patients with lumbar spondylolysis, which was treated conservatively in our institute from 2014 to 2019. We extracted data from cases in high school students and younger children who were diagnosed with fresh unilateral spondylolysis with bone marrow edema by MRI at the first visit. We excluded cases in which acute lumbar spondylolysis occurred on both sides, and cases in patients who did not consent to conservative therapy.

### Methods

Conservative therapy included using a rigid brace, stopping exercising (including physical class at their school) and physiotherapy until the bone marrow edema disappeared as seen by MRI. Bony union as evaluated with reconstructed sagittal and axial CT images at the time of disappearance of bone marrow edema was the primary outcome of the present study. Failure of bony union was defined as bone incontinuity in reconstructed sagittal and axial CT images.

To determine the factors significantly associated with failure of bony union, we conducted a univariate analysis with bony union at the final visit as the dependent variable and the following items as independent variables: sex, age, pathological stage of spondylolysis in the axial plane of CT [11], contralateral pseudarthrosis, lesion level, and spina bifida occulta. The disease stage was classified as progressive or not (prelysis and early stage) and the level of pathology was separated into L5 and non-L5. Variables with *p* < 0.1 were included in logistic regression analysis (forward–backward stepwise selection method). Any item with *p* < 0.05 was defined as a factor associated with bony union failure.

## Result

We included data from 92 patients with cases of unilateral spondylolysis on MRI. Successful bony union was achieved in 66 patients after conservative treatment and the remaining 26 patients failed to obtain bony union, which was diagnosed as pseudarthrosis. The patients included 72 boys and 20 girls with an average age of 14.4 (range 9–18) years. A prelysis stage was found in 21 patients, early stage was found in 50, and progressive stage in 21. Contralateral pseudarthrosis was found in 25 patients, whereas no pseudarthrosis was found in the remaining 67. The level of spondylolytic lesion was L3 in 9 patients, L4 in 19, and L5 in 64 (Table 1). There were 54 patients with spina bifida occulta.

**Table 1:** Patient demographics

Patient Demographics (*n* = 92)	
Treatment Result	
Union	66
Pseudoarthrosis	26
Sex	
Male	72
Female	20
Age at First Visit (years old)	14.4 (9-18)
Pathological Stage	
Prelysis	21
Early	50
Progressive	21
Contralateral Condition	
Normal	67
Bone defect	25
Vertebral Level of The Lesion	
L3	9
L4	19
L5	64
Intercurrent SBO	
With	54
Without	38

Univariate analysis revealed that progressive pathological stage (*p* < 0.001), contralateral pseudarthrosis (*p* < 0.001), and L5 lesion level (*p* = 0.001) were significantly associated with bony union failure, whereas age (*p* = 0.40), sex (*p* = 0.85), and spina bifida occulta (*p* = 0.20) were not (Table 2). The 3 factors showing *p* < 0.1 in univariate analysis were then analyzed using logistic regression with a forward–backward stepwise selection method. The logistic regression revealed that progressive pathological stage (*p* = 0.004, odds ratio (OR) 20.0; 95% CI 3.0–193.9), L5 lesion level (*p* = 0.002, OR 175; 95% CI 8.5–8192) and contralateral pseudarthrosis (*p* < 0.001, OR 78.8; 95% CI 13–846) (Table 3) were significantly associated with failure of bony union.

**Table 2:** Univariate analysis

Univariate analysis	*p*-value (#: *p*<0.1)
Age	0.4
Sex	0.85
L5 or non-L5	0.001#
Spina Bifida Occulta	0.2
CT Prgoressive Stage	<0.001#
Contralateral Pseudarthrosis	<0.001#

## Discussion

In the present study, we found that lesion level (L5), pathological stage, and contralateral pseudarthrosis were significantly associated with failure of bone union in patients with acute unilateral lumbar spondylolysis and bone marrow edema.

L5 is the most frequent lesion level for lumbar spondylolysis [12]. The union rate of L5 lesions is lower than that for other levels [13, 14]. In the present study, we also found that the union rate at the vertebral level of L5 was significantly lower than that for other vertebral levels. The progressive stage of the disease was previously reported to have a significant negative impact on bony union [15], because according to the stage of disease progression a larger bony defect occurs, which may inhibit bony union.

**Table 3:** Stepwise Logistic Regression

Stepwise Logistic Regression	*p*-value (*: *p*<0.05)	odds ratio (95% CI)
L5 or non-L5	0.002*	175 (8.5-8192)
CT Prgressive Stage	0.004*	20.0 (3.0-193.9)
Contralateral Pseudarthrosis	<0.001*	78.8 (13-846)

**Fig. 1:**
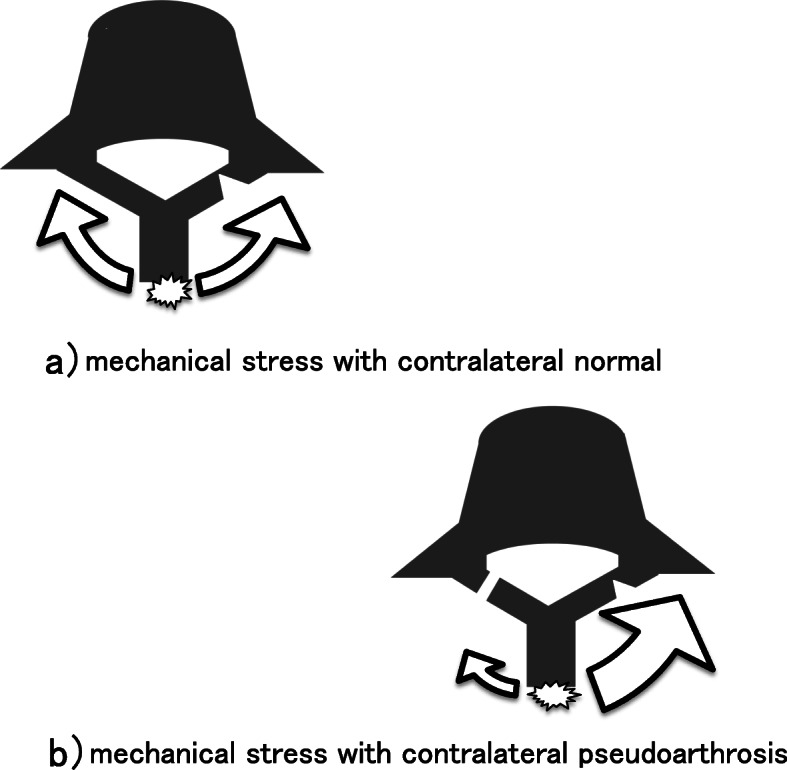
The direction and magnitude of the mechanical load are indicated by arrows. **a** In the normal case without contralateral spondylolysis, the mechanical stress from the posterior is evenly distributed to the left and right vertebral arches. **b** When a bone defect occurs due to terminal stage spondylolysis, the mechanical stress from the posterior is concentrated at the site of acute spondylolysis where bone continuity remains. Therefore, stability is not attained and the union rate is low

In addition to the abovementioned known factors that have a significant negative impact on bony union after conservative therapy for lumbar spondylolysis, we demonstrated that unilateral lumbar spondylolysis with contralateral pseudarthrosis is a major risk factor for bony union failure, with an odds ratio of 78.8. Mechanical load can increase significantly when there is pseudarthrosis on the contralateral side of the vertebral arch, resulting in the development of additional fresh spondylolysis [8, 16]. Because contralateral pseudarthrosis disrupts the bone continuity of the vertebral arch, bony union of fresh spondylolysis may be inhibited by the concentration of stress, possibly resulting in a lower rate of bony union (Fig. 1).

In the case of fatigue fractures of the extremities, delayed treatment can lead to bony union failure, resulting in a poor prognosis. Therefore, early surgery is indicated according to the prognosis of the stress fractures related to the site and/or stage of the fracture. Surgery may also be considered for stress fractures to shorten rest period and hasten the return to the sports, although they can be healed by conservative treatment. This principal can be indicated for lumbar spondylolysis. As is widely known, conservative treatment is the first choice for fresh lumbar spondylolysis associated with bone marrow edema, and surgery might be considered only when low back pain persists [9, 17].

According to the present results, conservative therapy aimed at bony union should not be applied automatically to all cases. Conservative therapy for acute unilateral spondylolysis should be considered carefully and the patients fully informed of risk factors for bony union failure including progerssive stage, a lesion level at L5, and contralateral pseudarthrosis. In such cases, an option is to conduct symptomatic treatment without prolonged external fixation and rest, without aiming for bony union. Another option is to perform direct surgical repair to avoid long rest for attaining bony union earlier.

The limitation of this study is that detailed clinical findings of each case such as the degree of pain could not be confirmed. In addition, it is a retrospective survey and the details of the case where the treatment was dropped out could not be confirmed.

## Conclusions

Conservative therapy aiming at bony union is contraindicated in cases of acute unilateral spondylolysis when the pathological stage is progressive, the lesion level is L5, or there is contralateral pseudarthrotic spondylolysis.

## Data Availability

The datasets generated during and analyzed during the current study are available from the corresponding author on reasonable request.

## Abbreviation

L5: the 5th lumbar spinal vertebra
